# β2-Microglobulin is a Novel and Reliable Biomarker for Predicting Ischemic Stroke Recurrence: A Prospective Cohort Study

**DOI:** 10.3389/fphar.2022.916769

**Published:** 2022-06-17

**Authors:** Fu-yong Hu, Wentao Wu, Qiuwan Liu, Juncang Wu, Hualing Guo, Jing Yang, Zhuqing Wu, Ke Jiang, Guoping Wang, Yu Qian, Wei Ge, Sen Qun

**Affiliations:** ^1^ School of Public Health, Bengbu Medical College, Bengbu, China; ^2^ Division of Life Sciences and Medicine, The Stroke Center and Department of Neurology, The First Affiliated Hospital of USTC, University of Science and Technology of China, Hefei, China; ^3^ Department of Neurosurgery, Beijing Tiantan Hospital, Capital Medical University, Beijing, China; ^4^ Department of Neurology, The Hefei Affiliated Hospital of Anhui Medical University (No. 2 People’s Hospital of Hefei), Hefei, China; ^5^ Department of Neurosurgery, The First Affiliated Hospital of Anhui Medical University, Hefei, China; ^6^ Department of Neurology, The Affiliated Hospital of Xuzhou Medical University, Xuzhou, China

**Keywords:** β2-microglobulin, acute ischemic stroke, recurrence, immunity and inflammation, propensity score matching

## Abstract

Immune and inflammatory mechanisms play key roles in the development and outcome of acute ischemic stroke (AIS). β2-Microglobulin (β2M) is the light chain of major histocompatibility complex-1 (MHC-1), which can directly and quickly reflect the immune and inflammatory state of the body. Previous studies have shown a close relationship between β2M and AIS, but its relationship with the recurrence of AIS has not been reported. This study attempted to explore the relationship between β2M and the recurrence of AIS. A single-center AIS cohort involving 135 patients was followed for approximately 26–46 months. Clinical and laboratory data from the patients were collected when hospitalized. The endpoint was the occurrence of recurrent AIS after patients were discharged. Propensity score matching was used to match cohort groups. Cox regression analysis was used to predict risk factors for recurrent AIS, and receiver operating characteristic curve (ROC) analysis was used to calculate the optimal cutoff value for discriminating recurrence in patients with AIS. The rate of recurrence was 29.6% [95% CI, 21.8%–37.3%] in the follow-up group. Patients with higher levels of serum β2M had a higher risk of AIS recurrence than patients with lower levels of β2M (adjusted hazard ratio, 3.214 [95% CI, 1.557–6.633]; adjusted hazard ratio after matching, 5.831, [95% CI, 2.052–16.572]). A β2M value of 2.31 mg/L was calculated by ROC analysis as the optimal cutoff value for AIS recurrence (area under the curve 0.770, [95% CI, 0.687–0.853]). As a quick responder to the body’s immune and inflammatory states, β2M may be a novel and reliable biomarker in predicting AIS recurrence.

## Introduction

Ischemic stroke (IS) is the leading cause of death and disability among adults worldwide (O'Donnell et al., 2016). With the aging of society and the acceleration of urbanization, the burden of IS in our country has exploded, and it has become an important public health problem that seriously affects the national economy and people’s livelihoods. In parallel with its high incidence, IS currently lacks effective prevention and treatment (Wu et al., 2019). Therefore, the etiology and pathology of IS have become the focus of research in recent years.

Chronic inflammation and an aberrant immune response are characteristic features of atherosclerosis, a leading cause of cardiovascular diseases and IS (Solanki et al., 2018). Additionally, immunity and inflammation are key factors in the pathobiology of AIS. For example, the inflammatory signaling pathway is associated with the ischemic cascade, and stroke is associated with immunosuppression and infection (Iadecola and Anrather, 2011). Immunity and inflammation are considered to be the core pathological mechanisms of IS.

β2-microglobulin (β2M) is the light chain of major histocompatibility complex-1 (MHC-1), a small-molecular-weight protein of 11.8 kD that is secreted by nucleated cells. β2M is an important structural protein by which CD8^+^ T lymphocytes regulate host immune recognition of self and nonself antigens as well as immunoglobulin transport (Nomura et al., 2014) and is closely associated with the innate and adaptive immune systems (Ardeniz et al., 2015), possibly being a potential initiator of inflammatory responses (Xie and Yi, 2003).

There are many reports about the relationship between β2M and peripheral organ diseases (including tumors) related to immunity and inflammation (Wilson et al., 2007; Josson et al., 2011; Hermansen et al., 2012; Mao et al., 2020), and the central nervous system (CNS) has traditionally been regarded as immune-privileged, though this has been questioned (Piehl and Lidman, 2001). The mechanism of β2M and poststroke immunity and inflammation may be complex, and there are relatively few reports.

Previous studies have found that high levels of β2M were associated with an increased risk of IS among women (Rist et al., 2017). Our previous work also found that serum β2M levels are positively correlated with an increased risk of AIS and adverse prognosis, and are strongly associated with the risk score for IS recurrence (Essen Stroke Risk Score, ESRS) (Hu et al., 2019; Qun et al., 2019). However, due to the lack of long-term follow-up, its relationship with IS recurrence has not been specifically studied. In this study, we conducted a detailed analysis of the relationship between β2M and the recurrence of cerebral infarction through 26–46 months of follow-up.

## Materials and Methods

### Ethical Statements

This study was approved by the Research Ethics Committee of the First Affiliated Hospital of the University of Science and Technology of China (USTC). Written informed consent was obtained from all participants and their guardians while involved in this study.

### Participant Recruitment

Patients admitted to the hospital for AIS were recruited from September 2015 to July 2017 and diagnosed as per their medical history, symptoms and signs, and diffusion-weighted magnetic resonance imaging (DWI). The patients were treated with antiplatelet drugs and statin therapy. Anticoagulant therapy was given for cardiac embolism and etiological treatment was given for other determined strokes. All patients were discharged in a stable condition.

The exclusion criteria for participants were as follows (O'Donnell et al., 2016): serious systemic diseases, such as acute or chronic renal dysfunction, or endocrine diseases (other than diabetes mellitus) (Wu et al., 2019); the use of immunosuppressant drugs (steroids) (Solanki et al., 2018); the presence of cancer (Iadecola and Anrather, 2011), trauma (Nomura et al., 2014), infectious diseases, or (Ardeniz et al., 2015) hematological disorders.

### Laboratory Assays and Clinical Information

We collected blood samples for laboratory tests in the morning (between 6:00 and 7:00 a.m.) following an overnight fast. Serum β2M was tested by the particle-enhanced turbidimetric immunoassay method. The intra-assay coefficient of variation ranged from 2.4 to 3.8%, meanwhile, the inter-assay coefficient of variation ranged from 1.7 to 2.2%. C-reactive protein (CRP) was tested by the immune transmission turbidity method. Other biochemical parameters, including fasting blood glucose (GLU), homocysteine (Hcy), creatinine (Crea), urea nitrogen (BUN), uric acid (UA), low-density lipoprotein cholesterol (LDL), triglyceride (TG), total cholesterol (CHOL), high-density lipoprotein cholesterol (HDL), very low-density lipoprotein cholesterol (VLDL), cystatin C (CysC), were measured by enzymatic method. All serum biochemical parameters were assayed by an automatic biochemical analyzer (HITACHI Automatic Analyzer 7600-020, Japan). Laboratory personnel was blinded to clinical data as well as clinicians to laboratory data.

Information on patient demographic characteristics, including age, sex, stroke risk factors for hypertension referenced by systolic blood pressure (SBP) or diastolic blood pressure (DBP), type 2 diabetes, stroke history, coronary heart disease (CHD), and smoking and alcohol consumption was collected.

ESRS and the National Institute of Health Stroke Scale (NIHSS) were used to evaluate the patient situation (Weimar et al., 2009), (Lyden et al., 1994). The Trial of ORG 10172 in Acute Stroke Treatment (TOAST) was used to classify the five subtypes of acute ischemic stroke: large-artery atherosclerosis (LAA), cardiac embolism (CE), small-artery occlusion (SAO), a stroke of other determined cause (SOC), and stroke of undetermined cause (SUC) (Chen et al., 2012).

### Endpoint

The participants were followed up after discharge from the hospital until the following events occurred: AIS, death, and loss to follow-up. The deadline was set on 26 July 2019.

### Propensity Score Matching

**FIGURE 1:**
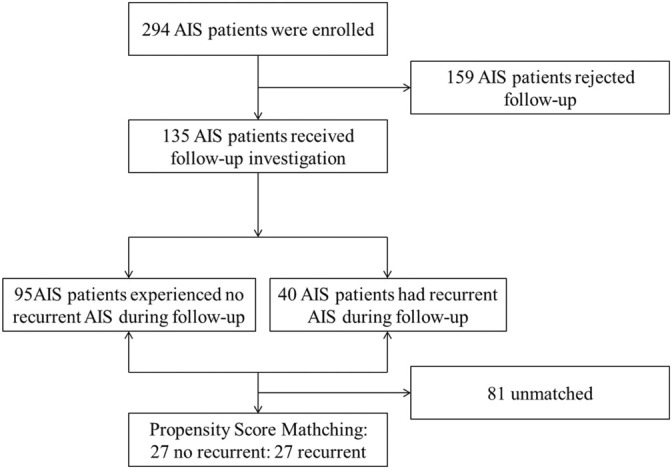
Data attrition flowchart.

The propensity score was calculated for each patient based on a multivariable logistic regression model. We only included age and CHD according to the significant differences in demographic characteristics between recurrent and no recurrent groups. A 1:1 ratio using a greedy nearest neighbor method with a tolerance of 0.02 was performed. Figure 1 presents the participants selection.

### Statistical Analysis

Statistical analysis was conducted with the Statistical Package for the Social Sciences version (SPSS) 22.0 (IBM Corp., Armonk, NY, USA). Quantitative data were tested for normality using the Shapiro–Wilk test. Variables that followed a normal distribution are expressed as mean ± standard deviation or as the median and interquartile range (IQR). Categorical variables are presented as frequencies and percentages. Differences between recurrent and no recurrent groups were assessed by the independent-samples *t* test, the Mann–Whitney *U*-test, the Chi-square test, or Fisher’s exact test, as appropriate. The optimal cutoff value of β2M for stroke recurrence was evaluated by the ROC analysis. Cox regression was used to develop a recurrence risk model.

### Results

A total of 294 AIS patients were enrolled in this study, among those discharged patients, 135 received follow-up investigation, and 159 (54.1%) rejected follow-up. Table 1 shows baseline characteristics of no follow-up group and follow-up group, and there were no significant differences between the two groups (*p* > 0.05).

**TABLE 1:** The characteristics between no follow-up group and follow-up group.

Variable	No follow-up group	Follow-up group	Statistics	*p* value
Male, n (%)	92 (57.9)	78 (57.8)	χ2 = 0.000	0.988
Age (years)	72 (16)	71 (15)	Z = -0.508	0.612
Hypertension, n (%)	109 (73.2)	96 (71.1)	χ2 = 0.147	0.701
Type 2 diabetes, n (%)	34 (22.8)	32 (23.7)	χ2 = 0.031	0.860
Stroke history, n (%)	50 (33.8)	43 (32.1)	χ2 = 0.091	0.762
CHD, n (%)	29 (19.6)	25 (18.7)	χ2 = 0.040	0.842
Smoker, n (%)	25 (17.0)	28 (20.9)	χ2 = 0.693	0.405
Alcohol user, n (%)	19 (12.9)	22 (16.4)	χ2 = 0.686	0.407

Up to the deadline, a total of 40 patients had recurrent AIS, and the rate of recurrence was 29.6% [95% CI, 21.8%–37.3%]. The recurrence rates were 0% [95% CI, 0.0%–7.0%] at 3 months, 6.7% [95% CI, 2.5%–10.9%] at 6 months, 17.0% [95% CI, 10.7%–23.3%] at 12 months, 23.7% [95% CI, 16.5%–30.9%] at 2 years, and 28.1% [95% CI, 20.6%–35.6%] at 3 years (Table 2). By propensity score matching, 27 no recurrent and 27 recurrent patients were matched.

**TABLE 2:** Rate of ischemic stroke recurrence (N = 135).

Month	N	Rate (95% CI)
0–3	0	0.0% (0.0%–7.0%)
0–6	9	6.7% (2.5%–10.9%)
0–12	23	17.0% (10.7%–23.3%)
0–24	32	23.7% (16.5%–30.9%)
0–36	38	28.1% (20.6%–35.6%)

### Comparison of Patients With AIS Recurrence and Those With no Recurrence

Compared to the patients with no recurrence, those with recurrence had higher age, an increased prevalence of CHD, and increased levels of Crea, BUN, CysC, CRP, β2M, NIHSS, and ESRS (*p* < 0.05). Other variables were not significantly different between the two groups (Table 3).

**TABLE 3:** Clinical and laboratory findings in recurrent and no recurrent patients.

Variable	Recurrent	No recurrent	Statistics	*p* value
	(N = 40)	(N = 95)		
Male, n (%)	27 (67.5%)	55 (57.9%)	χ2 = 1.089	0.297
Age (years)	73.8 ± 8.9	67.9 ± 12.2	t = 2.767	0.006
Hypertension, n (%)	32 (80.0%)	66 (69.5%)	χ2 = 1.568	0.211
Type 2 diabetes, n (%)	10 (25.0%)	22 (23.2%)	χ2 = 0.053	0.818
Stroke history, n (%)	17 (42.5%)	28 (29.5%)	χ2 = 2.149	0.143
CHD, n (%)	13 (32.5%)	9 (9.5%)	χ2 = 10.941	<0.001
Smoker, n (%)	9 (22.5%)	20 (21.1%)	χ2 = 0.035	0.852
Alcohol user, n (%)	5 (12.5%)	16 (16.8%)	χ2 = 0.404	0.525
SBP (mmHg)	150.1 ± 22.1	150.0 ± 22.1	t = 0.007	0.994
DBP (mmHg)	84.7 ± 16.9	83.9 ± 15.4	t = 0.265	0.791
Hcy (μmol/L)	13.2 (7.0)	11.7 (5.0)	Z = 1.889	0.059
GLU (mmol/l)	5.81 (1.60)	5.75 (1.67)	Z = 0.099	0.921
Crea (μmol/L)	81.55 ± 20.67	71.45 ± 17.44	t = 2.904	0.004
BUN (mmol/L)	6.48 ± 1.66	5.57 ± 1.49	t = 2.762	0.007
UA (μmol/L)	355.58 ± 108.65	332.62 ± 94.51	t = 1.232	0.220
LDL (mmol/L)	2.33 ± 0.64	2.33 ± 0.74	t = 0.039	0.969
TG (mmol/L)	1.30 (1.18)	1.38 (1.23)	Z = 0.800	0.424
CHOL (mmol/l)	4.13 ± 0.80	4.30 ± 0.97	t = -0.979	0.329
HDL (mmol/L)	1.35 ± 0.32	1.44 ± 0.36	t = -1.336	0.184
VLDL (mmol/L)	0.26 (0.24)	0.28 (0.25)	Z = -0.769	0.442
CysC (mg/L)	1.24 ± 0.40	1.08 ± 0.34	t = 2.286	0.024
CRP (mg/L)	0.98 (2.97)	0.52 (0.73)	Z = 2.166	0.0 30
β2M (mg/L)	2.58 ± 0.64	2.01 ± 0.52	t = 5.437	<0.001
NIHSS score	5 (7)	3 (3)	Z = 2.741	0.006
ESRS	3 (1)	2 (1)	Z = 3.779	<0.001
TOAST	-	-	-	0.194^a^
LAA	15 (37.5%)	30 (31.6%)	-	-
CE	9 (22.5%)	10 (10.5%)	-	-
SAO	16 (40.0%)	50 (52.6%)	-	-
SOC	0 (0.0%)	1 (1.1%)	-	-
SUC	0 (0.0%)	4 (4.2%)	-	-

a Fisher’s exact test.

### β2M is a Reliable Predictive Risk Factor for Recurrent AIS

**FIGURE 2:**
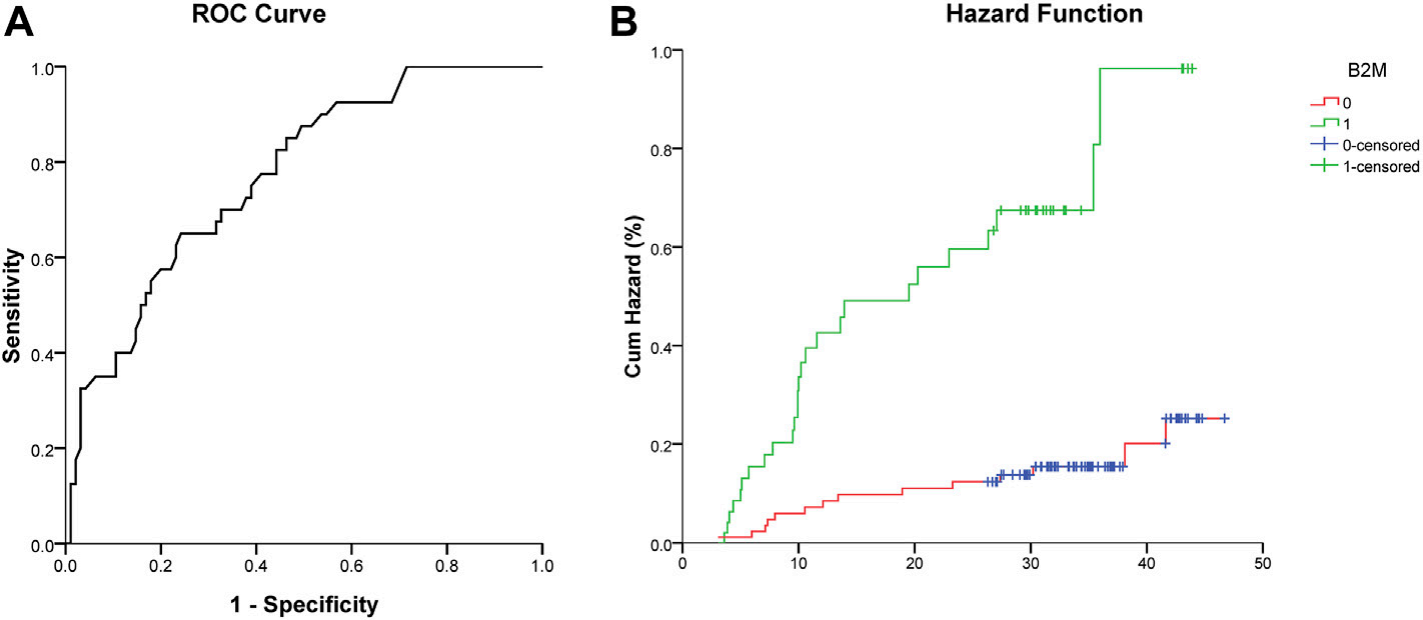
**(A)** ROC curve analysis showed a predictive serum level of β2M for stroke recurrence. **(B)** Cumulative hazard (%) of recurrent stroke between the low (0, β2M ≤ 2.31 mg/L) and high (1, β2M > 2.31 mg/L) β2M groups (Log-rank test, χ2 = 23.840, *p* < 0.001).

A β2M value of 2.31 mg/L was calculated by ROC analysis as the optimal cutoff value for AIS recurrence (area under the curve 0.770 [95% CI, 0.687–0.853]). The cutoff value had a sensitivity of 65.0% and a specificity of 75.8% (Figure 2A).

A Cox regression hazard model was used to predict the risk factors for recurrent AIS. The possible variables tested by univariate analysis (Table 3) were put into this model. Patients with higher levels of serum CRP (hazard ratio (HR) 1.033 [95% CI, 1.013–1.053]), β2M (HR 3.214 [95% CI, 1.557–6.633]), and attacked with CHD (HR 3.693 [95% CI, 1.614–8.453]) had higher risks of recurrent AIS, even after adjustments for other variables. The Kaplan–Meier curve showed the same result for β2M (Figure 2B). Variables including age, Crea, BUN, and CysC were not significantly different in terms of recurrent AIS (Table 4).

**TABLE 4:** Predictive risk factors for recurrent AIS using Cox proportional hazard regression.

	Model 1			Model 2		
	HR	95% CI (HR)	*p* value	HR	95% CI (HR)	*p* value
Age	1.008	0.974–1.044	0.642	1.019	0.983–1.057	0.296
CHD	2.972	1.363–6.478	0.006	3.693	1.614–8.453	0.002
Crea	1.012	0.988–1.036	0.323	1.006	0.981–1.032	0.648
BUN	0.927	0.713–1.205	0.569	0.981	0.753–1.278	0.889
CysC	0.519	0.198–1.362	0.183	0.566	0.200–1.600	0.283
CRP	1.027	1.009–1.045	0.003	1.033	1.013–1.053	0.001
β2M	3.105	1.579–6.103	0.001	3.214	1.557–6.633	0.002

Cox regression, method: Enter. Model 1: unadjusted. Model 2: adjusted by sex, hypertension, diabetes, stroke history, smoking, and alcohol consumption.

### Comparison of Patients With AIS Recurrence and Those With no Recurrence After Propensity Score Matching

Table 5 shows that after propensity score matching, compared to patients with no recurrence, those with recurrence had a higher level of Hcy, Crea, CRP, and β2M (*p* < 0.05), and other variables were not a significant difference. In the multivariable Cox proportional hazard regression model, Table 6 shows that only a higher level of β2M indicated a higher risk of AIS recurrence (HR 5.831, [95% CI, 2.052–16.572]).

**TABLE 5:** Clinical and laboratory findings in recurrent and no recurrent patients after propensity score matching.

Variable	Recurrent (N = 27)	No recurrent (N = 27)	Statistics	*p* value
Male, n (%)	20 (74.1%)	13 (48.1%)	χ2 = 3.818	0.051
Age (years)	72.5 ± 8.7	72.5 ± 8.7	t = 0.000	1.000
Hypertension, n (%)	21 (77.8%)	18 (66.7%)	χ2 = 0.831	0.362
Type 2 diabetes, n (%)	8 (29.6%)	7 (25.9%)	χ2 = 0.092	0.761
Stroke history, n (%)	10 (37.0%)	9 (33.3%)	χ2 = 0.081	0.776
CHD, n (%)	4 (14.8%)	4 (14.8%)	χ2 = 0.000	1.000
Smoker, n (%)	7 (25.9%)	1 (3.7%)	-	0.050^a^
Alcohol user, n (%)	4 (14.8%)	1 (3.7%)	-	0.351^a^
SBP (mmHg)	151.8 ± 22.5	147.9 ± 22.5	t = 0.640	0.525
DBP (mmHg)	85.9 ± 17.1	83.5 ± 12.9	t = 0.585	0.561
Hcy (μmol/L)	13.3 (9.0)	11.2 (5.0)	Z = 2.336	0.019
GLU (mmol/l)	6.25 (1.89)	5.98 (1.35)	Z = 0.329	0.742
Crea (μmol/L)	83.33 ± 21.57	67.89 ± 18.04	t = 2.854	0.006
BUN (mmol/L)	6.47 ± 1.61	5.79 ± 1.19	t = 1.792	0.079
UA (μmol/L)	348.00 ± 87.07	309.70 ± 110.91	t = 1.411	0.164
LDL (mmol/L)	2.16 ± 0.70	2.42 ± 0.65	t = 1.393	0.170
TG (mmol/L)	1.33 (0.95)	1.45 (0.75)	Z = 1.055	0.291
CHOL (mmol/l)	4.20 ± 0.83	4.18 ± 0.93	t = 0.076	0.940
HDL (mmol/L)	1.34 ± 0.36	1.45 ± 0.33	t = -1.141	0.259
VLDL (mmol/L)	0.27 (0.19)	0.29 (0.15)	Z = 1.030	0.303
CysC (mg/L)	1.22 ± 0.41	1.14 ± 0.29	t = 0.829	0.412
CRP (mg/L)	1.32 (3.05)	0.53 (0.73)	Z = 2.145	0.032
β2M (mg/L)	2.55 ± 0.58	1.90 ± 0.44	t = 4.606	<0.001
NIHSS score	4 (7)	3 (4)	Z = 1.047	0.295
ESRS	3 (1)	3 (2)	Z = 1.013	0.311
TOAST	-	-	-	0.054^a^
LAA	12 (44.4%)	4 (14.8%)	-	-
CE	3 (11.1%)	2 (7.4%)	-	-
SAO	12 (44.4%)	18 (66.7%)	-	-
SOC	0 (0.0%)	1 (3.7%)	-	-
SUC	0 (0.0%)	2 (7.4%)	-	-

a Fisher’s exact test.

**TABLE 6:** Predictive risk factors for recurrent AIS using Cox proportional hazard regression after propensity score matching.

	HR	95% CI (HR)	*p* value
Hcy	0.995	0.947–1.046	0.847
Crea	0.988	0.960–1.016	0.392
CRP	1.012	0.974–1.051	0.541
β2M	5.831	2.052–16.572	0.001

## Discussion

Ten risk factors are associated with 90% of the risk of AIS (O'Donnell et al., 2010), and nine out of 10 AIS are due to modifiable risk factors (Boehme et al., 2017). Thus, IS prevention has generally focused on modifiable risk factors. Hypertension, dyslipidemia, diabetes, smoking, alcohol consumption, air pollution, diets low in fruit and vegetables, and high sodium intake are the most common and modifiable risk factors for AIS in China (Guan et al., 2017; Wang et al., 2017). In view of these traditional risk factors, a series of prevention strategies have been developed. However, the incidence of IS is still expected to increase in China, and recurrence of IS may be the main cause (Del Brutto et al., 2019).

AIS survivors have a high risk of recurrence, and recurrent IS patients make up nearly one-third of all AIS (Bushnell et al., 2009; Pennlert et al., 2014). Recurrent AIS causes substantially higher morbidity and mortality than first-time AIS (Aarnio et al., 2014; Pennlert et al., 2014). Therefore, the prediction of recurrent IS may be a key strategy for IS prevention and treatment. Rapid identification of etiology is important for the prediction of recurrence of IS. Due to the complex etiology of ischemic stroke, reliable biomarkers of AIS recurrence are the focus of our attention.

In our cohort study, we found a strong correlation between plasma β2M levels and recurrence of AIS. After 26–46 months of follow-up, we found that a high level of serum β2M had nearly three times the risk of recurrent AIS (HR 3.105, 95% CI, 1.579–6.103) by means of the Cox proportional hazard regression model, suggesting that β2M is a reliable predictive risk factor for recurrent AIS. A β2M value of 2.31 mg/L was calculated by ROC analysis as the optimal cutoff value for stroke recurrence. We used propensity score matching to control the potential confounders. Considering the sample size, we only included covariates of age and CHD. After matching, the basic characteristics had no difference between the two groups, especially the NIHSS score. The result of Cox regression after matching still showed the positive relationship between β2M and recurrence of AIS.

Different from the above traditional risk factors, β2M is closely related to the innate and adaptive immune systems (Ardeniz et al., 2015), in addition, may be an initiator of inflammation (Xie and Yi, 2003). Atherosclerosis is a chronic inflammatory disease, and a previous study showed that β2M was independently and significantly associated with adverse cardiovascular outcomes in patients with prevalent asymptomatic carotid atherosclerosis (Amighi et al., 2011). Serum β2M may reflect levels of chronic inflammation in the body and thus serve as a risk marker for IS. However, the entire course of disease surrounding the occurrence, development, and outcome of IS is a vast array of immune-stimulating and inflammatory events (Iadecola and Anrather, 2011; Solanki et al., 2018). The serum level of β2M in our cohort may reflect a combination of two determinants: the basal level of the body and the emergency response state of the acute stage of AIS. As the basal state of the body’s immunity and inflammation, chronic inflammation is the core factor in the formation mechanism of atherosclerosis and the main cause of cardiovascular and cerebrovascular diseases (Diomedi et al., 2005; Ferrucci and Fabbri, 2018). Additionally, activation of both innate and adaptive immunity actively contributes to the initiation and progression of atherogenesis, from early endothelial dysfunction to the development of acute thrombotic complications triggered by plaque rupture or erosion (Ruparelia et al., 2017). On the other hand, AIS events are a concentrated expression process of immune stress and the inflammatory response: the immune system participates in the brain damage produced by ischemia. Subsequently, the brain actively promotes immunosuppression to avoid further brain damage but may promote stroke-associated infection and increase the mortality rate. Finally, regeneration and repair of ischemic brain tissue are regulated by adaptive immunity (Iadecola and Anrather, 2011). Serum β2M levels are also a concentrated reflection of immune and inflammatory states associated with post-AIS. As previously mentioned, IS-related immunity and inflammation are the results of the expression of systemic immune and inflammatory states before, during, and after stroke events. The physiological characteristics of β2M just meet the above requirements, so it can reflect the prognosis of stroke from the essential mechanism level of AIS. These studies provide a good explanation for β2M as a novel biological indicator of AIS recurrence. In the future, we will conduct relevant studies on β2M as a target to intervene in the recurrence of ischemic stroke, especially the relationship between β2M and the modifiable risk factors of AIS.

### Limitations

First, the relatively low number of research subjects is an important limitation of this study. Second, many patients are lost to follow-up. Third, data from multiple medical centers are needed to confirm our results. In the future, we will increase the sample size and reduce the loss of follow-up rate and conduct a multicenter case-control study to overcome the limitations of the current study.

## Conclusions

Following an AIS cohort of 26–46 months, we found that as a rapid responder of the immune and inflammatory state of the body, the level of peripheral blood β2M in patients with AIS has an important predictive value for the recurrence of AIS, suggesting that β2M is a novel and reliable predictor of recurrent AIS.

## Data Availability

The raw data supporting the conclusion of this article will be made available by the authors, without undue reservation.
